# Allvar Gullstrand: The Only Ophthalmologist Who Won the Nobel Prize

**DOI:** 10.7759/cureus.62513

**Published:** 2024-06-17

**Authors:** Abdelaziz A Awad, Hamad A Alkorbi, Hashem Abu Serhan

**Affiliations:** 1 Ophthalmology, Faculty of Medicine, Al-Azhar University, Cairo, EGY; 2 Internal Medicine, Qatar University, Doha, QAT; 3 Ophthalmology, Hamad Medical Corporation, Doha, QAT

**Keywords:** slit lamp, historical vignette, schematic eye model, nobel prize, ophthalmoscope, allvar gullstrand

## Abstract

Allvar Gullstrand, the Swedish ophthalmologist and Nobel laureate, was a self-taught mathematician who applied mathematics and higher-order equations to understand the optic system. His inventions, the slit lamp, and the ophthalmoscope are used in clinical practice for the diagnosis of eye diseases. With his efforts, he explained the accommodation, the process of changing the shape of the lens to focus on near or distant objects. In 1911, he was awarded the Nobel Prize in Physiology or Medicine. In 1913, he was elected as the first president of the Swedish Ophthalmological Society. In 1927, he was awarded the Graefe Medal of the Deutsche Ophthalmologische Gesellschaft.

## Introduction and background

**Figure 1:**
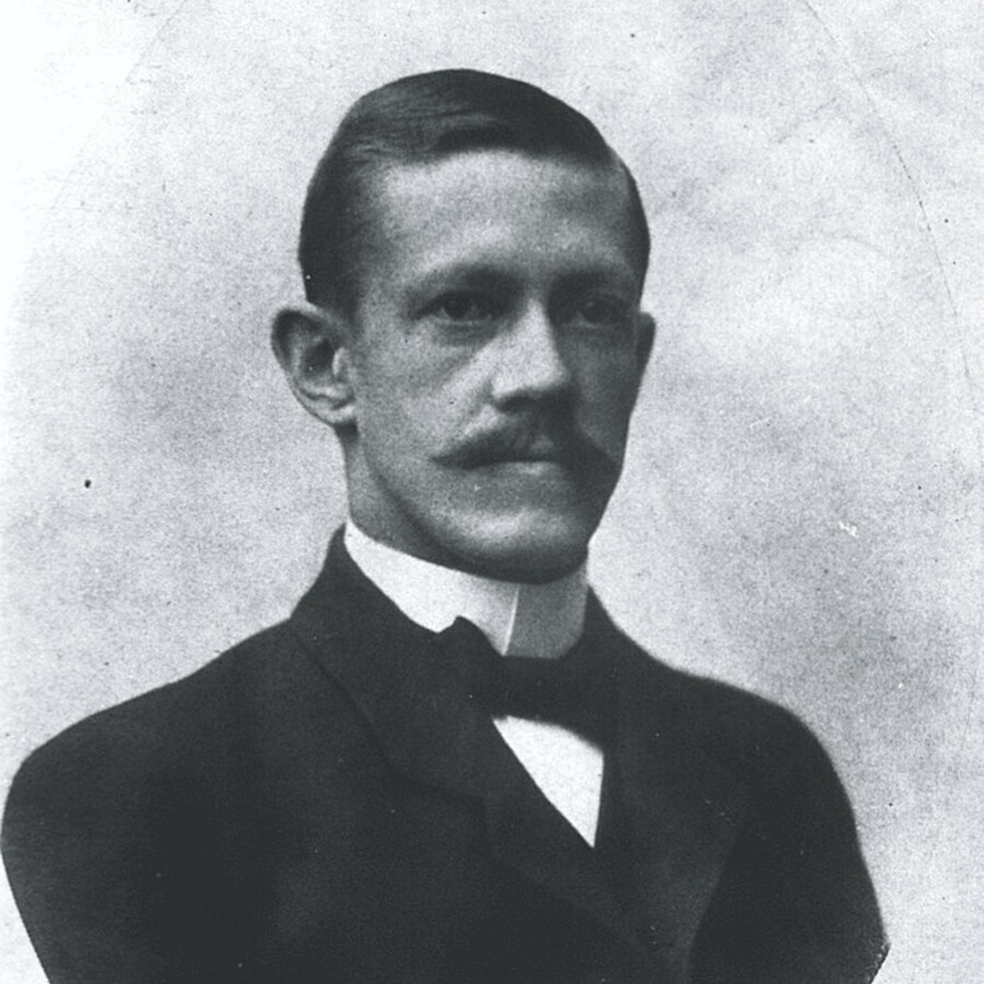
Allvar Gullstrand, MD (1862 – 1930) (Bain News Service, publisher). This image is available from the United States Library of Congress's Prints and Photographs division under the digital ID ggbain.06386. CC Public Domain Mark 1.0

Allvar Gullstrand (1862-1930) was a Swedish mathematician and the only ophthalmologist who won a Nobel Prize in Physiology or Medicine in 1911 due to his great contribution to the ophthalmology field. He also was part of the Nobel Committee for Physics from 1911 to 1929, serving as chairman from 1923 to 1929. In 1913, he was elected as the first president of the Swedish Ophthalmological Society. In 1922, he attended the International Congress of Ophthalmology in Washington, DC, as the Swedish representative, where he demonstrated the slit lamp. In 1927, he was awarded the Graefe Medal of the Deutsche Ophthalmologische Gesellschaft (Figure 1) [1].

## Review

His life

Allvar Gullstrand was born on 5, 1862, in Landskrona, Sweden. After finishing the study of mathematics at Jonkoping in 1880, he started studying medicine in Uppsala. In 1885, he traveled to Vienna for years and married Signe Christine Breitholtz, and they had a daughter who died of diphtheria. He completed his training in Stockholm in 1888. His published thesis in 1888, entitled ‘A Contribution to the Theory of Astigmatism’, was considered the foundation for the subsequent work that made him remembered every year [2-4]. In 1930, he had a cerebral hemorrhage, leading to his death.

His inventions

**Figure 2:**
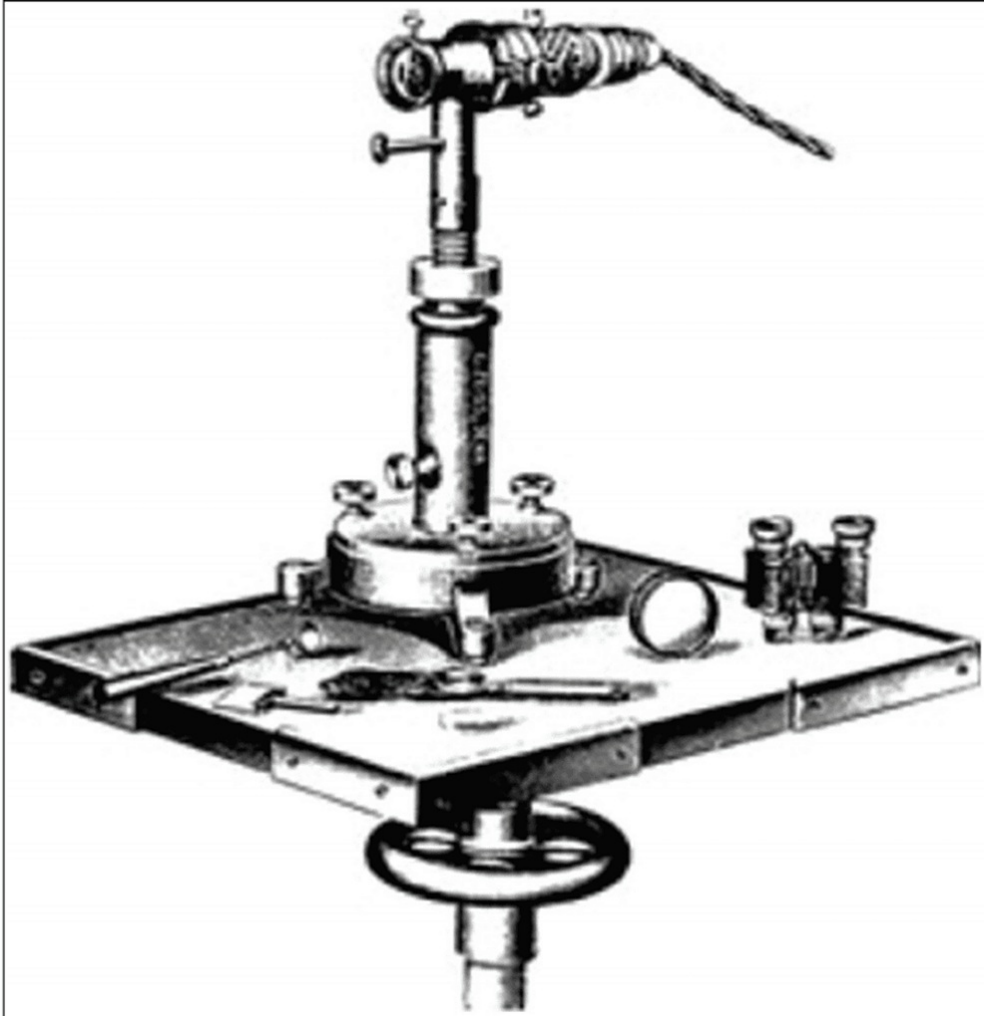
The slit lamp. Source: [2] (This is an open-access article which is distributed under the terms of the Creative Commons Attribution‑NonCommercial‑ShareAlike 4.0 License)

He announced the first model of the slit-lamp as an eye illuminator at the Heidelberg Ophthalmology Congress in 1911, which was not realized until 1916. He used the basis of the electric bulb developed by Walter Nernst (Figure 2). The slit lamp was then improved, and versatility increased. The combination of the binocular corneal microscope and slit lamp that was designed for the Zeiss factory in Jena by Siegfried Czapski increased the accessibility of elucidating the ocular diseases’ finer changes by every ophthalmologist. His results got slow international acceptance as he published his work in Swedish. He continued working on astigmatism, performing higher orders of equation terms. The required constant number of characteristics to describe a bundle of monochromatic light that emerges from a source of point light was found to be 10. Also, he developed the reflexless ophthalmoscope, which reduced glare from the cornea, making it easier to examine the cornea. Much of his work was founded on mathematical models and equations he created to better understand the function of the eye. He designed a schematic eye that included the lens curvature, the distance between the lens and the cornea, and the refractive indexes of all components [1,2].

Accommodation explanation

The increased convexity of the anterior surface of the lens due to ciliary muscle contraction and zonular relaxation accounts for two-thirds of accommodation, which is called extracapsular in Gullstrand's terminology, while the resting one-third is intra-capsular [1,2]. Initially, Gullstrand was not able to foresee the ability of a slit lamp to help in diagnosis, measuring the corneal curvature, and studying the structural changes of the lens. The corneal rectangular appearance is slightly curved: Bowman’s membrane and the corneal epithelium form the anterior line, while the posterior line is formed by Descemet’s membrane, sandwiching the homogenous marbled stroma. Although the aqueous humour is clear in nature, extraneous particles can be seen moving in its thermal currents, such as bundles of blood cells. The cortex of the lens is enclosed by the anterior and posterior capsules and its innermost nucleus. The cellular nuclei's paucity and the close alignment between these cells can affect lens transparency. Also, increased levels of insoluble proteins and water can lead to the development of cataracts. Behind the lens, the anterior vitreous is clear. Variations on the anterior surface of the iris are easily observed, and the detection of defects in its pigmented posterior surface is possible [5,6].

Nobel Prize

Gullstrand was nominated for the Nobel Prize in Physics or Medicine in 1911 due to his work on the dioptric apparatus of the eye, and he declined the prize. As a member of the member of the physics committee, the deliberations of each committee are secret, so we do not know if he was present [7]. In 1910, when Albert Einstein was proposed for the Nobel Prize in physics, his relativity theory was met with arguments that remained for several years until the end of the war. Gullstrand, as a Nobel committee member, also argued against Einstein getting the Nobel Prize. He maintained his argument, as Einstein's theory was just a belief, which was not proven enough and not of the greatest utility for mankind.

Positions

Due to his love for science, he refused to be a vice chancellor of Uppsala University, a surgeon general of the Swedish army, or a president of the Swedish Government Board of Health. Also, he refused to go to Stockholm's Karolinska Institute for the same reasons. The academic senate of the University of Uppsala created a personal chair for him in physiological and physical optics to help him focus only on his research. He served on the medical Swedish board as an ophthalmology lecturer in 1891. He was the first ophthalmology professor at Uppsala in 1894.

## Conclusions

Allvar Gullstrand (1862-1930) was a Swedish ophthalmologist whose research into the physiological and geometric optics of the eye challenged existing theories by revealing new ways to examine the structures of the eye. His most notable invention was the slit lamp, but he was also responsible for the development of the reflex-free ophthalmoscope and schematic eye. He received the Nobel Prize in Medicine or Physiology in 1911.

## References

[1] Ehinger B, Grzybowski A (2011). Allvar Gullstrand (1862-1930)--the gentleman with the lamp. Acta Ophthalmol.

[2] Sen M, Honavar SG (2021). Allvar Gullstrand: prize and prejudice. Indian J Ophthalmol.

[3] Davison J, Wilson G (2005). Allvar Gullstrand. Clin Exp Ophthalmol.

[4] Ravin JG (2001). Allvar Gullstrand. Ophthalmology.

[5] Esteve-Taboada JJ, Montés-Micó R, Ferrer-Blasco T (2018). Schematic eye models to mimic the behavior of the accommodating human eye. J Cataract Refract Surg.

[6] Kaur K, Gurnani B (2023). Slit-lamp biomicroscope. StatPearls [Internet].

[7] Ravin JG (1999). Gullstrand, Einstein, and the Nobel Prize. Arch Ophthalmol.

